# Five New Species of *Marquandomyces* (Clavicipitaceae, Ascomycota) from Asia

**DOI:** 10.3390/jof11030180

**Published:** 2025-02-25

**Authors:** Lu-Yao Peng, Yi-Fan Wang, He Song, Islomjon Urinboev, Wen-Ying Zhuang, Yusufjon Gafforov, Xin-Cun Wang

**Affiliations:** 1 State Key Laboratory of Mycology, Institute of Microbiology, Chinese Academy of Sciences, Beijing 100101, China; pengluyao327@163.com (L.-Y.P.); zhuangwy@im.ac.cn (W.-Y.Z.); 2 University of Chinese Academy of Sciences, Beijing 100049, China; 3 College of Life Science, Hebei University, Baoding 071002, China; 4 School of Biotechnology, Jiangnan University, Wuxi 214122, China; yfwang.bai@foxmail.com; 5 Department of Nutrition and Health, China Agricultural University, Beijing 100083, China; 6 College of Modern Agriculture and Ecological Environment, Heilongjiang University, Harbin 150080, China; 13104631380@163.com; 7 Department of Botany, Biotechnology and Ecology, Faculty of Natural Sciences, Fergana State University, Ferghana 150100, Uzbekistan; 8 Central Asian Center of Development Studies, New Uzbekistan University, Tashkent 100007, Uzbekistan; 9 Department of Ecology, Faculty of Biology and Ecology, National University of Uzbekistan, Tashkent 100174, Uzbekistan

**Keywords:** Hypocreales, *Metarhizium*, *Paecilomyces*, phylogeny, taxonomy, tungsten mine

## Abstract

*Marquandomyces* is a recently established genus in the Clavicipitaceae and previously comprised only two known species. This study expands the understanding of its generic concept and species diversity through comprehensive fungal diversity surveys conducted from soil samples in China and Uzbekistan. As a result, five new species were identified based on morphological characterization and molecular phylogenetic analyses, and their detailed descriptions and illustrations are provided. This study emphasizes the unique ecological roles and specific habitats of these fungi and contributes to a relatively deep understanding of the genus *Marquandomyces* as well as its ecological significance.

## 1. Introduction

*Marquandomyces* Samson, Houbraken & Luangsa-ard, a monotypic genus, was established to accommodate *Paecilomyces marquandii* (Massee) S. Hughes [1]. This species has been extensively reported to play multiple crucial functions in agriculture and environmental remediation. It is an effective biocontrol agent against nematodes, notably reducing root galling in tomato [2] and increasing the head weights of lettuce [3]. It is also an important phosphate solubilizer to release phosphorus from iron and calcium phosphates, which in turn promotes the growth of crops, especially in alkaline soils [4,5,6,7]. Moreover, this species efficiently takes part in not only zinc and lead biosorption but also herbicide degradation, highlighting its potential applications in the elimination of heavy metals [8,9,10] and herbicides [11,12,13] from polluted areas.

The species *Verticillium marquandii* Massee was originally described by the British mycologist Massee in 1898. Hughes transferred it into the genus *Paecilomyces* Bainier in 1951, which was widely followed for a long time. Samson erected a new section, *Isarioidea*, in *Paecilomyces* and placed *P. marquandii* in it [14]. Phylogenetic analyses based on 18S rDNA revealed the polyphyletic nature of *Paecilomyces sensu lato*, and the members are distributed across two subclasses; the type species *P. variotii* Bainier and its thermophilic relatives belong to the order Eurotiales, while mesophilic species are located in the order Hypocreales (Clavicipitaceae and Hypocreaceae). *Paecilomyces marquandii* of the latter group is a member of Clavicipitaceae [15]. Further analyses inferred from the β-tubulin gene and ITS rDNA cannot determine its taxonomic position in the family [16]. A subsequent inference based on a five-gene dataset placed *P. marquandii* in the *Cordyceps taii* clade and revealed its close relationship with *Metarhizium* [17]. Then, it was transferred to the genus *Metarhizium*, although it is anamorphic and mononematous (not producing synnemata) [18]. This treatment has been accepted by some researchers [19] but not by others [20]. Therefore, a new genus, *Marquandomyces*, was proposed to accommodate this phylogenetically and ecologically special species [1]. In China, this species was first recorded in 2003 [21], and the second member of the genus was recently reported in the southwest part of the country [22].

In this study, the fungal diversity was investigated in soil samples collected from several provinces of China and the Western Tian Shan Mountains of Uzbekistan. As a result, five new species of *Marquandomyces* were discovered and reported.

## 2. Materials and Methods

### 2.1. Fungal Materials

Cultures were isolated, using the classical spread plate method, from soil samples collected from the Hebei and Heilongjiang provinces of China and the Tashkent Province of Uzbekistan from 2023 to 2024. The collection site in Hebei Province of China is in the North China Plain, with a warm-temperate climate, broad-leaved trees, and an altitude of 50 m; that in Heilongjiang Province of China is in the Northeast Plain, with a middle-temperate climate, coniferous and broad-leaved mixed forest, and an altitude of 100 m; and that in Tashkent Province of Uzbekistan is in the Western Tian Shan Mountains, with an altitude of 1800 m. Dried cultures were deposited in the Herbarium Mycologicum Academiae Sinicae (HMAS, Beijing, China), and the living ex-type strains were preserved in the China General Microbiological Culture Collection Center (CGMCC, Beijing, China).

### 2.2. Morphological Observations

Four standard growth media were used: Czapek yeast autolysate agar (CYA, yeast extract Oxoid, Hampshire, UK), malt extract agar (MEA, Amresco, Solon, OH, USA), oatmeal agar (OA), and potato dextrose agar (PDA). The methods for inoculation, incubation, microscopic examinations, and digital recordings followed our previous studies [23,24,25,26,27,28,29,30].

### 2.3. DNA Extraction, PCR Amplification, and Sequencing

DNA was extracted from cultures grown on PDA for 7 days using the Plant Genomic DNA Kit (DP305, TIANGEN Biotech, Beijing, China). Polymerase chain reaction (PCR) amplification of the internal transcribed spacer (ITS), large subunit (LSU), and translation elongation factor 1-α (TEF) genes was conducted using routine methods [31]. The products were purified and subject to sequencing on an ABI 3730 DNA Sequencer (Applied Biosystems, Foster, CA, USA).

### 2.4. Phylogenetic Analyses

Forward and reverse sequences newly generated in this study were assembled using Seqman v. 7.1.0 (DNASTAR Inc., Madison, WI, USA). The assembled sequences were deposited at GenBank. The sequences used for phylogenetic analyses are listed in Table 1. The sequences of each of the three single-gene datasets (ITS, LSU, and TEF) and the concatenated one were aligned using MAFFT v. 7.221 [32], then manually edited and concatenated in BioEdit v. 7.1.10 [33] and MEGA v. 11.0.13 [34]. Maximum likelihood (ML) analyses were conducted using RAxML-HPC2 [35] on ACCESS v. 8.2.12 via CIPRES Science Gateway v. 3.3 [36], with the default GTRCAT model and bootstrap (BP) iteration setting. Bayesian inference (BI) analyses were performed with MrBayes v. 3.2.7 [37]. Appropriate nucleotide substitution models and parameters were determined using Modeltest v. 3.7 [38]. Four MCMC chains were run for at least one million generations, and posterior probability (PP) values were estimated with the remaining 75% of trees after the burn-in phase. The consensus trees were viewed in FigTree v. 1.4.4 [http://tree.bio.ed.ac.uk/software/figtree/ (accessed on 28 December 2023)]. Species of *Purpureomyces* Luangsa-ard, Samson & Thanakitp. served as outgroups.

## 3. Results

To reconstruct the phylogeny of *Marquandomyces*, the single-gene datasets (ITS, LSU, and TEF) and the concatenated one were compiled and analyzed. The detailed characteristics of the datasets are listed in Table 2.

**Figure 1:**
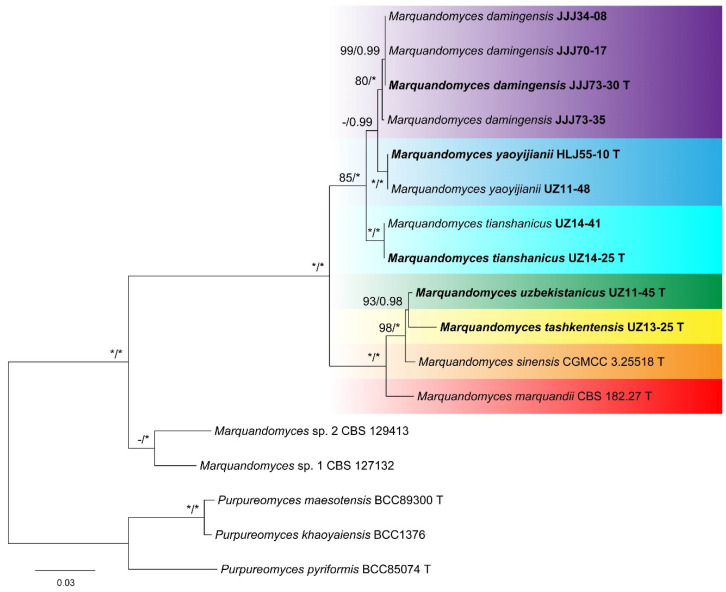
Maximum likelihood phylogeny of *Marquandomyces* inferred from the concatenated ITS, LSU, and TEF datasets. Bootstrap values ≥ 70% (**left**) or posterior probability values ≥ 0.95 (**right**) are indicated at nodes. Asterisk denotes 100% bootstrap or 1.00 posterior probability.

The phylogeny of *Marquandomyces* inferred from the concatenated dataset is shown in Figure 1. Nine species, including two unnamed ones, were grouped into three clades. One of them included four species, i.e., the type of the genus *M. marquandii*; *M. sinensis*; and two new ones, UZ11-45 and UZ13-25, both from Uzbekistan. Another contained three to-be-described species: (1) strains JJJ34-08, JJJ70-17, JJJ73-30, and JJJ73-35, all from Hebei Province, China, (2) HLJ55-10 from Heilongjiang Province, China, and UZ11-48 from Uzbekistan; (3) UZ14-25 and UZ14-41 from Uzbekistan. The last clade consisted of two unnamed species, *M.* sp. 1 CBS 127132 and *M.* sp. 2 CBS 129413.

The topology of the ITS tree was similar to that of the concatenated one, except that strain JJJ73-35 did not cluster with the other three (Figure S1). In the LSU phylogeny of the genus, the five to-be-described new species were clearly determined, but *M.* sp. 1 CBS 127132 clustered with HLJ55-10 and UZ11-48, and *M.* sp. 2 CBS 129413 was closely related to JJJ73-35 (Figure S2). In the TEF tree, the five new species were differentiated with strong supports, while the positions of the two unnamed species were different from the concatenated phylogeny: *M.* sp. 1 CBS 127132 similarly clustered with HLJ55-10 and UZ11-48, but *M.* sp. 2 CBS 129413 was closely related to UZ14-25 and UZ14-41 (Figure S3).

## 4. Taxonomy

***Marquandomyces*** Samson, Houbraken & Luangsa-ard, *Stud. Mycol.* **2020**, *95*, 194.

Type species: *M. marquandii* (Massee) Samson, Houbraken & Luangsa-ard, *Stud. Mycol.* **2020**, *95*, 194.

Colony characteristics: On CYA, MEA, OA, or PDA 25 °C, 14 days: Colonies nearly circular or irregular; mycelia white or colorless; texture velutinous, sometimes floccose or funiculose; conidia *en masse* purplish vinaceous or cinnamon-colored; reverse yellow. On CYA 37 °C, no growth.

Micromorphology: Hyphae branched, septate, hyaline, smooth. Conidiophores biverticillate or much branched, hyaline to light yellow-brown, smooth-walled. Phialides cylindrical to ellipsoidal, tapering into distinct neck. Conidia ellipsoidal to fusiform, smooth-walled to finely roughened, hyaline, *en masse* pale vinaceous.

Distribution: Worldwide. Reported from Europe (UK, France, Germany, Greece, Netherlands, Portugal, Russia), Asia (China, Republic of Korea, Uzbekistan, Vietnam), North America (USA), and South America (Brazil).

Habitat: In soil, occasionally on mushrooms.

**Figure 2:**
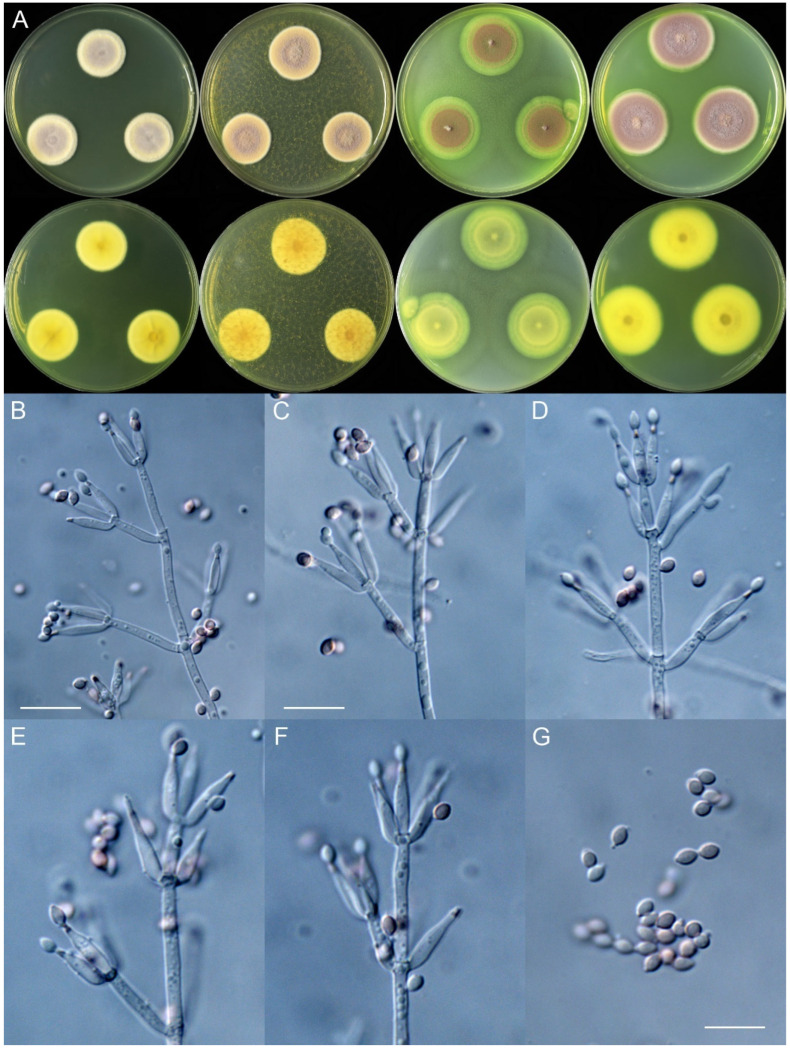
*Marquandomyces damingensis* (JJJ73-30). (**A**) Colonies: top row left to right, obverse CYA, MEA, OA, and PDA; bottom row left to right, reverse CYA, MEA, OA, and PDA; (**B**–**F**) Conidiophores; (**G**) Conidia. Bars: (**B**) = 15 µm; (**C**) = 12.5 µm, also for (**D**); (**G**) = 10 µm, also for (**E**–**F**).

***Marquandomyces damingensis*** X.C. Wang, L.Y. Peng & W.Y. Zhuang, sp. nov. Figure 2

Fungal Names: FN572184

Etymology: The specific epithet refers to the type locality.

Typification: China. Hebei Province, Handan City, Daming County, Dajie Town, Dajie Village, 36°17′32″ N 115°12′6″ E, in soil of winter wheat (*Triticum aestivum* L.) field, 12 February 2024, Xin-Cun Wang, culture, Yi-Fan Wang, JJJ73-30 (holotype HMAS 353201, ex-type strain CGMCC 3.28567).

DNA barcodes: ITS PQ484187, LSU PQ484201, TEF PQ469018.

Colony diam., 14 days, 25 °C (unless stated otherwise): CYA 22–30 mm; CYA 37 °C no growth; MEA 23–35 mm; OA 29–38 mm; PDA 20–36 mm.

Colony characteristics: On CYA 25 °C, 14 days: Colonies nearly circular or irregular, plain or protuberant, sometimes concave at centers, radially sulcate or not; margins narrow, entire, or restricted; mycelia white; texture velutinous; sporulation sparse to dense; conidia *en masse* purplish vinaceous; soluble pigments absent; exudates absent; reverse yellow-brown.

On MEA 25 °C, 14 days: Colonies nearly circular, plain, protuberant at centers; margins narrow to moderately wide, entire; mycelia white; texture velutinous, sometimes floccose or funiculose at the centers; sporulation dense; conidia *en masse* purplish vinaceous; soluble pigments absent; exudates absent; reverse yellow-brown.

On OA 25 °C, 14 days: Colonies nearly circular or irregular, plain, concentrically sulcate or not; margins narrow to wide, entire, or irregular; mycelia white or colorless; texture velutinous; sporulation sparse to moderately dense; conidia *en masse* purplish vinaceous; soluble pigments yellowish; exudates absent; reverse yellow-brown.

On PDA 25 °C, 14 days: Colonies nearly circular, plain, slightly protuberant at centers; margins narrow to moderately wide, entire or protuberant; mycelia white; texture velutinous; sporulation sparse to dense; conidia *en masse* purplish vinaceous; soluble pigments yellowish; exudates absent; reverse yellow-brown.

Micromorphology: Hyphae branched, septate, hyaline, smooth, 2.5–4.5 μm diam. Conidiophores terverticillate or biverticillate, hyaline, smooth-walled, 30–65 × 2.0–2.5 μm, with whorls of 2–6 phialides. Phialides cylindrical to ellipsoidal, tapering into distinct neck, 9.5–13 × 2.0–2.5 μm. Conidia ellipsoidal to fusiform, smooth-walled, hyaline, *en masse* pale vinaceous, 3.0–4.0 × 2.5–3.0 μm.

Additional strains examined: China. Hebei Province, Handan City, Daming County, Xiweizhuang Town, Diwang Square, 36°16′43″ N 115°7′59″ E, in soil of *Populus* forest, 15 July 2023, Xin-Cun Wang, culture, Yi-Fan Wang, JJJ34-08; *ibid.*, Yangqiao Town, G45 Zhang River Service Area, 36°24′50″ N 115°5′28″ E, in soil, 6 February 2024, Xin-Cun Wang, culture, Yi-Fan Wang, JJJ70-17; *ibid.*, Dajie Town, Dajie Village, 36°17′32″ N 115°12′6″ E, in soil of winter wheat (*Triticum aestivum* L.) field, 12 February 2024, Xin-Cun Wang, culture, Yi-Fan Wang, JJJ73-35.

Notes: This species is a sister to *M. yaoyijianii* based on the phylogeny of the genus (Figure 1). Molecularly, it differs from the latter in 8 bp for ITS, 2 bp for LSU, and 4 bp for TEF; morphologically, it produces much denser sporulation on MEA, YES, and PDA media and has much longer conidiophores (30–65 vs. 18–55 μm) than the latter. Intraspecific variations were observed among the four strains of the fungus. The strain JJJ73-35 differs from the type strain in 2 bp for ITS and 1 bp for LSU. Except JJJ73-35, the other three share the same molecular characteristics.

**Figure 3:**
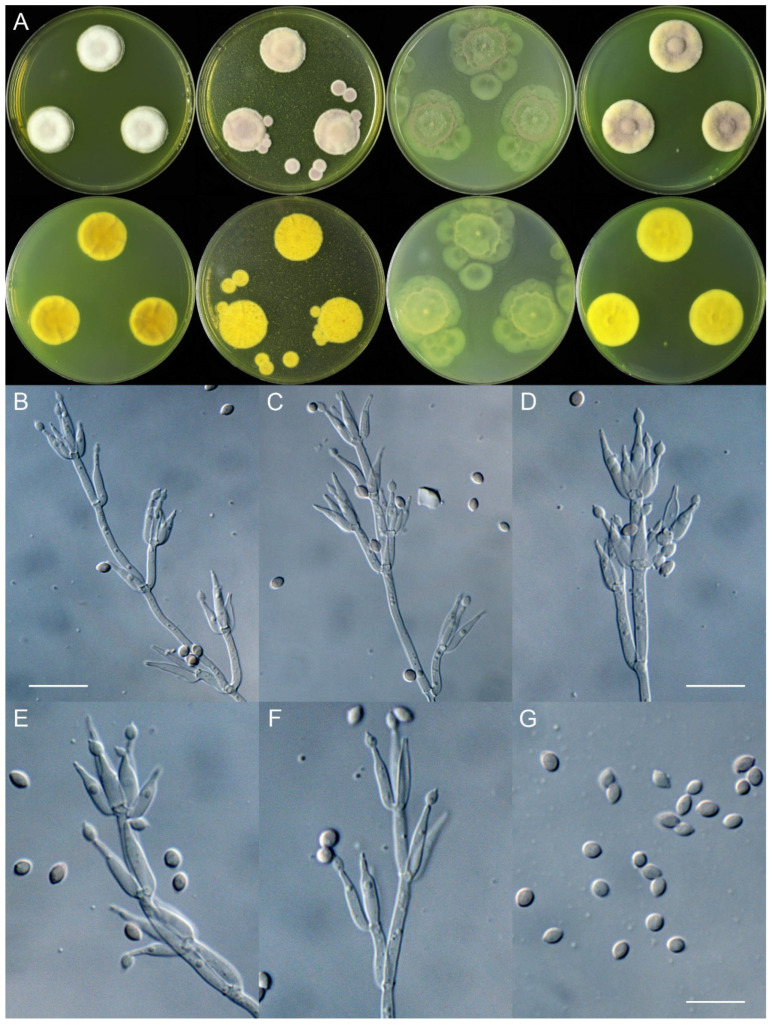
*Marquandomyces tashkentensis* (UZ13-25). (**A**) Colonies: top row left to right, obverse CYA, MEA, OA, and PDA; bottom row left to right, reverse CYA, MEA, OA, and PDA; (**B**–**F**) Conidiophores; (**G**) Conidia. Bars: (**B**) = 15 µm, also for (**C**); (**D**) = 12.5 µm; (**G**) = 10 µm, also for (**E**–**F**).

***Marquandomyces tashkentensis*** X.C. Wang, L.Y. Peng, Gafforov & W.Y. Zhuang, sp. nov. Figure 3

Fungal Names: FN572185

Etymology: The specific epithet refers to the type locality.

Typification: Uzbekistan. Tashkent Province, Parkent District, Chatkal State Biosphere Nature Reserve, Bashkizilsay area, Western Tian Shan (Tien Shan) Mountains, 41°10′21″ N 69°49′36″ E, in soil, 17 January 2024, Islomjon Urinboev & Yusufjon Gafforov, culture, Lu-Yao Peng, UZ13-25 (holotype HMAS 353202, ex-type strain CGMCC 3.28568).

DNA barcodes: ITS PQ484189, LSU PQ484203, TEF PQ469020.

Colony diam., 14 days, 25 °C (unless stated otherwise): CYA 23–24 mm; CYA 37 °C no growth; MEA 22–23 mm; OA 31–34 mm; PDA 25–26 mm.

Colony characteristics: On CYA 25 °C, 14 days: Colonies nearly circular, protuberant, concave at centers; margins narrow, entire; mycelia white; texture velutinous; sporulation sparse; conidia *en masse* with a tint of purplish vinaceous; soluble pigments greenish yellow; exudates absent; reverse yellow-brown.

On MEA 25 °C, 14 days: Colonies nearly circular, protuberant; margins narrow; mycelia white; texture velutinous; sporulation dense; conidia *en masse* purplish vinaceous; soluble pigments greenish yellow; exudates absent; reverse yellow-brown.

On OA 25 °C, 14 days: Colonies irregular, plain; margins wide, entire or irregular; mycelia colorless; texture velutinous; sporulation sparse; conidia *en masse* purplish vinaceous; soluble pigments greenish yellow; exudates absent; reverse greenish yellow.

On PDA 25 °C, 14 days: Colonies nearly circular, protuberant; margins narrow, entire; mycelia white; texture velutinous; sporulation moderately dense; conidia *en masse* purplish vinaceous; soluble pigments greenish yellow; exudates absent; reverse yellow-brown.

Micromorphology: Hyphae branched, septate, hyaline, smooth, 2.0–2.5 μm diam. Conidiophores quaterverticillate or terverticillate, hyaline, smooth-walled, 30–90 × 2.0–3.0 μm, with whorls of 2–7 phialides. Phialides cylindrical to ellipsoidal, tapering into distinct neck, 8.5–18 × 2.0–4.0 μm. Conidia ellipsoidal to fusiform, smooth-walled, hyaline, 3.0–4.0 × 2.5–3.0 μm.

Notes: This species is located in the subclade containing the type species of the genus and close to *M. uzbekistanicus* in the phylogeny (Figure 1). Molecularly, it differs from the latter in 19 bp for ITS, 4 bp for LSU, and 1 bp for TEF. In addition, compared with the type species of the genus, it differs in 27 bp for ITS, 14 bp for LSU, and 2 bp for TEF, and it is distinguished from *M. sinensis* in 21 bp for ITS, 7 bp for LSU, and 2 bp for TEF. Morphologically, it differs from *M. uzbekistanicus* in slower growth rates on the four media, conidia *en masse* lacking cinnamon color on PDA at 25 °C, conidiophores less branched, and longer phialides (8.5–18 vs. 7.5–13.5 μm).

**Figure 4:**
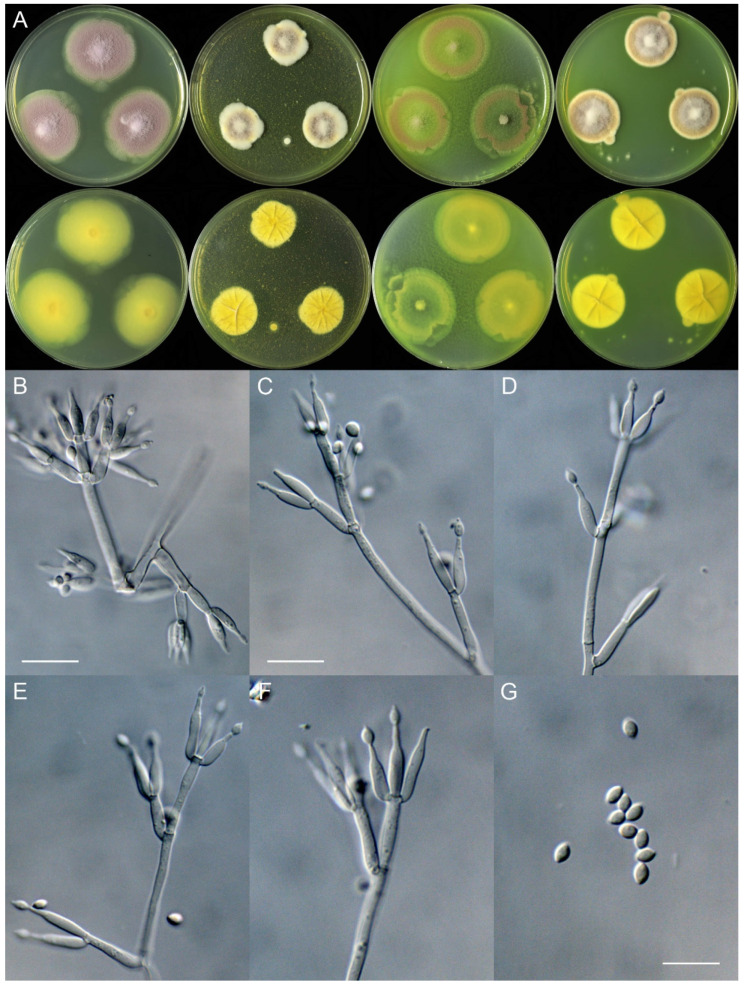
*Marquandomyces tianshanicus* (UZ14-25). (**A**) Colonies: top row left to right, obverse CYA, MEA, OA, and PDA; bottom row left to right, reverse CYA, MEA, OA, and PDA; (**B**–**F**) Conidiophores; (**G**) Conidia. Bars: (**B**) = 15 µm; (**C**) = 12.5 µm, also for (**D**,**E**); (**G**) = 10 µm, also for (**F**).

***Marquandomyces tianshanicus*** X.C. Wang, L.Y. Peng, Gafforov & W.Y. Zhuang, sp. nov. Figure 4

Fungal Names: FN572186

Etymology: The specific epithet refers to the type locality.

Typification: Uzbekistan. Tashkent Province, Parkent District, Chatkal State Biosphere Nature Reserve, Bashkizilsay area, Western Tian Shan (Tien Shan) Mountains, 41°10′33″ N 69°50′59″ E, in soil, 17 January 2024, Islomjon Urinboev & Yusufjon Gafforov, culture, Lu-Yao Peng, UZ14-25 (holotype HMAS 353203, ex-type strain CGMCC 3.28569).

DNA barcodes: ITS PQ484190, LSU PQ484204, TEF PQ469021.

Colony diam., 14 days, 25 °C (unless stated otherwise): CYA 38–40 mm; CYA 37 °C no growth; MEA 18–23 mm; OA 34–37 mm; PDA 26–30 mm.

Colony characteristics: On CYA 25 °C, 14 days: Colonies nearly circular, plain, protuberant at centers; margins moderately wide, entire or irregular; mycelia white; texture velutinous; sporulation dense; conidia *en masse* light purplish vinaceous; soluble pigments absent; exudates absent; reverse greenish yellow.

On MEA 25 °C, 14 days: Colonies nearly circular or irregular, protuberant; margins narrow, entire or protuberant; mycelia white; texture floccose to funiculose; sporulation moderately dense to dense; conidia *en masse* purplish vinaceous; soluble pigments greenish yellow; exudates absent; reverse yellow.

On OA 25 °C, 14 days: Colonies nearly circular or irregular, plain, protuberant at centers; margins moderately wide, entire or irregular; mycelia colorless; texture velutinous, floccose at centers; sporulation sparse to moderately dense; conidia *en masse* purplish vinaceous; soluble pigments greenish yellow; exudates absent; reverse greenish yellow.

On PDA 25 °C, 14 days: Colonies nearly circular, plain, protuberant at centers; margins narrow, entire; mycelia white; texture velutinous, floccose or funiculose at centers; sporulation dense; conidia *en masse* Mars yellow to purplish vinaceous; soluble pigments greenish yellow; exudates absent; reverse yellow-brown.

Micromorphology: Hyphae branched, septate, hyaline, smooth, 2.0–3.0 μm diam. Conidiophores terverticillate, occasionally quarterverticillate or biverticillate, hyaline, smooth-walled, 27–70 × 2.0–3.5 μm, with whorls of 2–6 phialides. Phialides cylindrical to ellipsoidal, tapering into distinct neck, 7.5–17 × 2.0–3.5 μm. Conidia ellipsoidal to fusiform, smooth-walled, hyaline, 3.0–4.0 × 2.0–3.0 μm.

Additional strain examined: Uzbekistan. Tashkent Province, Parkent District, Chatkal State Biosphere Nature Reserve, Bashkizilsay area, Western Tian Shan (Tien Shan) Mountains, 41°10′33″ N 69°50′59″ E, in soil, 17 January 2024, Islomjon Urinboev & Yusufjon Gafforov, culture, Lu-Yao Peng, UZ14-41.

Notes: In the phylogeny of the genus, *M. tianshanicus* clustered with *M. damingensis* and *M. yaoyijianii* (Figure 1). Molecularly, it differs from *M. damingensis* in 29 bp for ITS, 1 bp for LSU, and 6 bp for TEF, and it differs from *M. yaoyijianii* in 33 bp for ITS, 3 bp for LSU, and 6 bp for TEF. Morphologically, it differs from the latter two species in a faster growth rate on CYA at 25 °C and quarterverticillate conidiophores.

**Figure 5:**
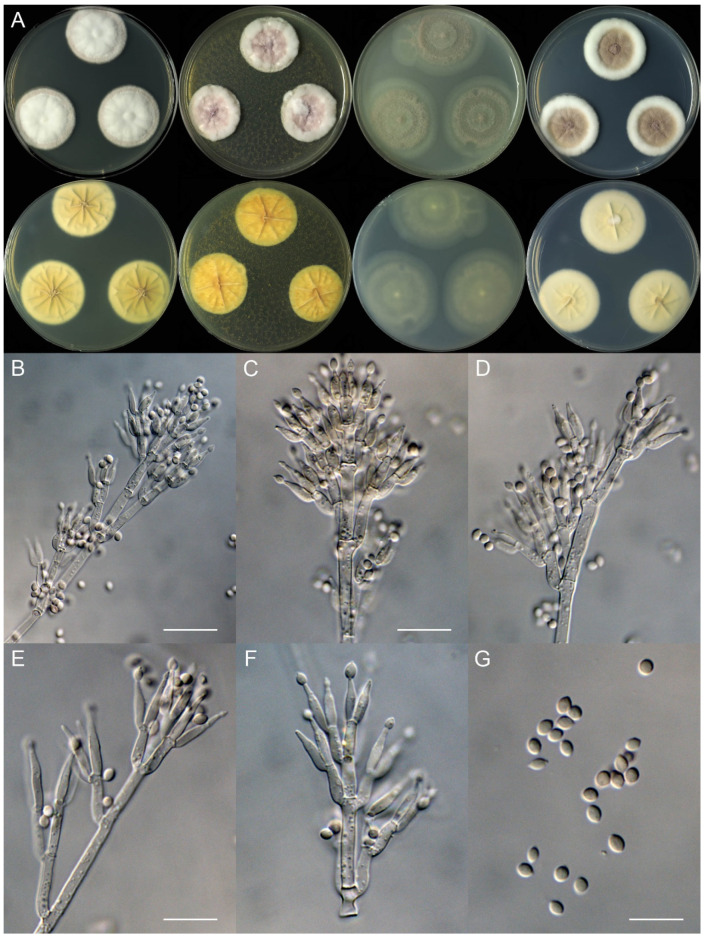
*Marquandomyces uzbekistanicus* (UZ11-45). (**A**) Colonies: top row left to right, obverse CYA, MEA, OA, and PDA; bottom row left to right, reverse CYA, MEA, OA, and PDA; (**B**–**F**) Conidiophores; (**G**) Conidia. Bars: (**B**) = 20 µm; (**C**) = 15 µm, also for (**D**); (**E**) = 12.5 µm, also for (**F**); (**G**) = 10 µm.

***Marquandomyces uzbekistanicus*** X.C. Wang, L.Y. Peng, Gafforov & W.Y. Zhuang, sp. nov. Figure 5

Fungal Names: FN572187

Etymology: The specific epithet refers to the type locality.

Typification: Uzbekistan. Tashkent Province, Parkent District, Chatkal State Biosphere Nature Reserve, Bashkizilsay area, Western Tian Shan (Tien Shan) Mountains, 41°10′30″ N 69°49′20″ E, in soil, 17 January 2024, Islomjon Urinboev & Yusufjon Gafforov, culture, Lu-Yao Peng, UZ11-45 (holotype HMAS 353204, ex-type strain CGMCC 3.28570).

DNA barcodes: ITS PQ484192, LSU PQ484206, TEF PQ469023.

Colony diam., 14 days, 25 °C (unless stated otherwise): CYA 33–34 mm; CYA 37 °C no growth; MEA 29–32 mm; OA 46–47 mm; PDA 33–34 mm.

Colony characteristics: On CYA 25 °C, 14 days: Colonies nearly circular, protuberant, concave at centers, radially sulcate; margins narrow, entire; mycelia white; texture velutinous; sporulation sparse; conidia *en masse* purplish vinaceous; soluble pigments absent; exudates absent; reverse yellow-brown.

On MEA 25 °C, 14 days: Colonies nearly circular, plain, radially sulcate; margins wide, protuberant; mycelia white; texture velutinous, floccose at margins; sporulation sparse to moderately dense; conidia *en masse* purplish vinaceous; soluble pigments absent; exudates absent; reverse yellow-brown.

On OA 25 °C, 14 days: Colonies nearly circular, plain; margins wide, entire; mycelia colorless; texture velutinous, floccose at centers; sporulation sparse; conidia *en masse* purplish vinaceous; soluble pigments absent; exudates absent; reverse pale.

On PDA 25 °C, 14 days: Colonies nearly circular, protuberant at margins, radially sulcate; margins wide, protuberant; mycelia white; texture velutinous; sporulation moderately dense; conidia *en masse* cinnamon-colored; soluble pigments absent; exudates absent; reverse white to buff.

Micromorphology: Hyphae branched, septate, hyaline to light yellow-brown, smooth, 2.5–4.0 μm diam. Conidiophores hexaverticillate, pentaverticillate, or quarterverticillate, hyaline to light yellow-brown, smooth-walled, 50–100 × 2.5–4.0 μm, with whorls of 2–5 phialides. Phialides cylindrical to ellipsoidal, tapering into distinct neck, 7.5–13.5 × 2.0–3.0 μm. Conidia ellipsoidal to fusiform, smooth-walled, hyaline, 2.5–3.5 × 2.0–2.5 μm.

Notes: This species is sister to *M. tashkentensis* (Figure 1) and its molecular distinctions from the latter were previously noted (see notes of *M. tashkentensis*). In addition, compared with the type species of the genus, it differs in 16 bp for ITS, 13 bp for LSU, and 1 bp for TEF; it is distinguished from *M. sinensis* in 20 bp for ITS, 3 bp for LSU, and 1 bp for TEF. Morphologically, it differs from *M. tashkentensis* in cinnamon conidia *en masse* on PDA at 25 °C, much more branching conidiophores, and shorter phialides.

**Figure 6:**
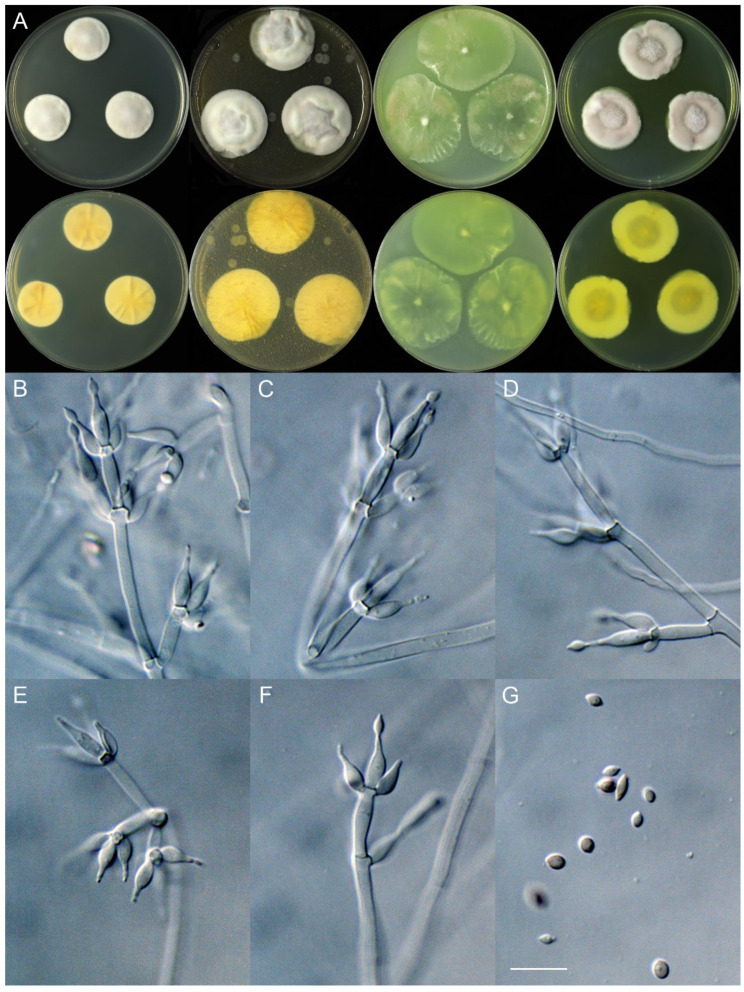
*Marquandomyces yaoyijianii* (HLJ55-10). (**A**) Colonies: top row left to right, obverse CYA, MEA, OA, and PDA; bottom row left to right, reverse CYA, MEA, OA, and PDA; (**B**–**F**) Conidiophores; (**G**) Conidia. Bars: (**G**) = 10 µm, also for (**B**–**F**).

***Marquandomyces yaoyijianii*** X.C. Wang, L.Y. Peng & W.Y. Zhuang, sp. nov. Figure 6

Fungal Names: FN572188

Etymology: The specific epithet is in honor of the Chinese mycologist Professor Yi-Jian Yao, who was born in Fujian Province in January 1955 and made contributions to the taxonomy of clavicipitaceous fungi. He is also the founder of the database Fungal Names, which is one of the three recognized repositories by the Nomenclature Committee for Fungi (NCF) for the registration of nomenclatural novelties.

Typification: China. Heilongjiang Province, Shuangyashan City, Lingdong District, Yangbishan iron-tungsten mine, 46°35′3″ N 131°3′51″ E, in soil of a wastewater pool, 13 May 2023, Xin-Cun Wang and He Song, culture, He Song, HLJ55-10 (holotype HMAS 353205, ex-type strain CGMCC 3.28571).

DNA barcodes: ITS PQ484193, LSU PQ484207, TEF PQ469024.

Colony diam., 14 days, 25 °C (unless stated otherwise): CYA 23–28 mm; CYA 37 °C no growth; MEA 30–36 mm; OA 40–49 mm; PDA 31–53 mm.

Colony characteristics: On CYA 25 °C, 14 days: Colonies nearly circular, protuberant or slightly protuberant, radially sulcate; margins narrow to wide, entire; mycelia white; texture velutinous; sporulation sparse; conidia *en masse* purplish vinaceous; soluble pigments absent; exudates absent; reverse yellow-brown.

On MEA 25 °C, 14 days: Colonies nearly circular or irregular, plain, protuberant at centers, radially sulcate or not; margins moderately wide, entire or irregular; mycelia white; texture velutinous; sporulation sparse to moderately dense; conidia *en masse* purplish vinaceous; soluble pigments absent; exudates absent; reverse yellow-brown.

On OA 25 °C, 14 days: Colonies nearly circular or irregular, plain, slightly protuberant at centers; margins moderately wide to wide, entire or irregular; mycelia white or colorless; texture velutinous; sporulation sparse; conidia *en masse* purplish vinaceous; soluble pigments absent; exudates absent; reverse pale to yellow.

On PDA 25 °C, 14 days: Colonies irregular, protuberant or plain; margins narrow to moderately wide, entire or irregular; mycelia white or colorless; texture velutinous, slightly floccose at centers; sporulation sparse to dense; conidia *en masse* purplish vinaceous; soluble pigments yellow or absent; exudates absent; reverse yellow-brown.

Micromorphology: Hyphae branched, septate, hyaline, smooth, 2.0–3.0 μm diam. Conidiophores terverticillate or biverticillate, hyaline, smooth-walled, 18–55 × 2.0–2.5 μm, with whorls of 2–5 phialides. Phialides cylindrical to ellipsoidal, tapering into distinct neck, 7–13.5 × 2.5–3.0 μm. Conidia ellipsoidal to fusiform, smooth-walled, hyaline, 3.0–5.0 × 2.0–3.0 μm.

Additional strain examined: Uzbekistan. Tashkent Province, Parkent District, Chatkal State Biosphere Nature Reserve, Bashkizilsay area, Western Tian Shan (Tien Shan) Mountains, 41°10′30″ N 69°49′20″ E, in soil, 17 January 2024, Islomjon Urinboev & Yusufjon Gafforov, culture, Lu-Yao Peng, UZ11-48.

Notes: This species is phylogenetically close to *M. damingensis* (Figure 1). Molecularly, it differs from the latter in 8 bp for ITS, 2 bp for LSU, and 4 bp for TEF; morphologically, it differs in sparser sporulation on MEA, YES, and PDA media and shorter conidiophores (18–55 vs. 30–65 μm).

**Key to species of *Marquandomyces***
1. Distributed worldwide
*M. marquandii*
1. Distributed in Asia22. Chlamydospore-like structures present
*M. sinensis*
2. Chlamydospore-like structures absent33. Conidia *en masse* on PDA 25 °C cinnamon-colored
*M. uzbekistanicus*
3. Conidia *en masse* on PDA 25 °C purplish vinaceous44. Sporulation on OA 25 °C dense54. Sporulation on OA 25 °C sparse65. Growth rate on CYA 25 °C fast (more than 35 mm)
*M. tianshanicus*
5. Growth rate on CYA 25 °C slow (no more than 30 mm)
*M. damingensis*
6. Growth rate on MEA 25 °C fast (no less than 30 mm)
*M. yaoyijianii*
6. Growth rate on MEA 25 °C slow (less than 25 mm)
*M. tashkentensis*



## 5. Discussion

Five new species of *Marquandomyces* were determined by multiple-gene phylogeny (Figure 1), which were also supported by the single locus analyses (Figures S1–S3). They were described and illustrated in detail, and their molecular and morphological distinctions were provided as well. These findings obviously updated our understanding of the genus and broadened our knowledge of the Clavicipitaceae.

The Tian Shan Mountains, also known as Tien Shan, cover a large mountain system situated in the Eurasia hinterland and are the furthest mountains from the sea. As one of the seven mountain systems in the world, the Tian Shan Mountains stretch through four countries (China, Kazakhstan, Kyrgyzstan, and Uzbekistan) with a length of 2500 km from east to west. Xinjiang Tianshan in China and Western Tien Shan in the other countries, both featuring diverse landscapes and home to exceptionally rich biodiversity, have been included in UNESCO’s World Heritage List. Four of the five new species in this work were isolated from this area, which proves that it is a biodiversity hotspot. This study marks the first record of the genus *Marquandomyces* in Uzbekistan and consequently from the Central Asian region. The findings significantly extend the geographical distribution of *Marquandomyces*, emphasizing Asia as an important hub of fungal biodiversity.

The type strain of *M. yaoyijianii* was isolated from an iron-tungsten mine in China. Diverse fungi have been discovered in iron mines, e.g., *Penicillium spinulosum* and *P. ubiquetum* from an American underground mine [39]. Although *Aspergillus niger* has been reported to be able to extract tungsten using a bioleaching technique [40], research on fungal communities from a tungsten mine is rare. China has the largest tungsten reserves and the fourth-largest iron reserves in the world. Fungal biodiversity and their functions in these mines remain unclear, and more efforts are badly needed.

Overall, this study provides valuable insights into the phylogenetic relationships, morphological diversity, and ecological roles of *Marquandomyces* species. Given the recent establishment of this genus and the limited research available, it is evident that *Marquandomyces* remains underexplored. The discovery of novel species in previously unsampled regions indicates that further surveys are essential to fully understand the global diversity of this genus. Targeted studies in other biogeographically distinct and understudied ecosystems could provide new insights into the evolutionary history and ecological roles of *Marquandomyces*. Additionally, exploring its ecological interactions, bioactive properties, and potential applications could reveal valuable resources for agriculture, environmental remediation, and biotechnology. This research highlights the importance of global collaboration in understanding the diversity and ecological significance of *Marquandomyces* and in harnessing its potential for sustainable use.

## Figures and Tables

**Table 1:** Species and sequences of *Marquandomyces* and *Purpureomyces* used in phylogenetic analyses.

Species	Strain	Locality	Substrate	ITS	LSU	TEF	Reference
***Marquandomyces damingensis*** X.C. Wang, L.Y. Peng & W.Y. Zhuang, sp. nov.	JJJ34-08	China: Hebei	soil	**PQ484185**	**PQ484199**	**PQ469016**	This study
	JJJ70-17	China: Hebei	soil	**PQ484186**	**PQ484200**	**PQ469017**	This study
	JJJ73-30 = CGMCC 3.28567 T	China: Hebei	soil	**PQ484187**	**PQ484201**	**PQ469018**	This study
	JJJ73-35	China: Hebei	soil	**PQ484188**	**PQ484202**	**PQ469019**	This study
*Marquandomyces marquandii* (Massee) Samson, Houbraken & Luangsa-ard 2020	CBS 182.27 T	USA: Iowa	soil	MH854923	EF468845	EF468793	[17,20]
*Marquandomyces sinensis* Zhi Y. Zhang & Y.F. Han 2024	CGMCC 3.2551 T	China: Guizhou	soil	OR680543	OR680607	OR858937	[22]
***Marquandomyces tashkentensis*** X.C. Wang, L.Y. Peng, Gafforov & W.Y. Zhuang, sp. nov.	UZ13-25 = CGMCC 3.28568 T	Uzbekistan: Tashkent	soil	**PQ484189**	**PQ484203**	**PQ469020**	This study
***Marquandomyces tianshanicus*** X.C. Wang, L.Y. Peng, Gafforov & W.Y. Zhuang, sp. nov.	UZ14-25 = CGMCC 3.28569 T	Uzbekistan: Tashkent	soil	**PQ484190**	**PQ484204**	**PQ469021**	This study
	UZ14-41	Uzbekistan: Tashkent	soil	**PQ484191**	**PQ484205**	**PQ469022**	This study
***Marquandomyces uzbekistanicus*** X.C. Wang, L.Y. Peng, Gafforov & W.Y. Zhuang, sp. nov.	UZ11-45 = CGMCC 3.28570 T	Uzbekistan: Tashkent	soil	**PQ484192**	**PQ484206**	**PQ469023**	This study
***Marquandomyces yaoyijianii*** X.C. Wang, L.Y. Peng & W.Y. Zhuang, sp. nov.	HLJ55-10 = CGMCC 3.28571 T	China: Heilongjiang	soil	**PQ484193**	**PQ484207**	**PQ469024**	This study
	UZ11-48	Uzbekistan: Tashkent	soil	**PQ484194**	**PQ484208**	**PQ469025**	This study
*Marquandomyces* sp. 1	CBS 127132	USA: Nebraska	soil	MT078882	MT078857	MT078849	[1]
*Marquandomyces* sp. 2	CBS 129413	USA: Wisconsin	soil	MT561567	MT078859	MT078851	[1]
*Purpureomyces khaoyaiensis* (Hywel-Jones) Luangsa-ard, Samson & Thanakitp. 2020	BCC1376	Thailand	Lepidoptera larva	KX983460	KX983462	KX983457	[19]
*Purpureomyces maesotensis* Luangsa-ard, Noisrip. Thanakitp. & Samson 2020	BCC89300 T	Thailand	Lepidoptera larva	MN781917	MN781876	MN781733	[1]
*Purpureomyces pyriformis* Luangsa-ard, Noisrip., Himaman, Mongkols. & Thanakitp. & Samson 2020	BCC85074 T	Thailand	Lepidoptera larva	MN781929	MN781873	MN781730	[1]

GenBank accession numbers in bold indicate the newly generated sequences.

**Table 2:** Detailed characteristics of the datasets.

Dataset	No. of Seq.	Length of Alignment (bp)	No. of Variable Sites	No. of Parsimony-Informative Sites	Model for BI
ITS	17	609	185	141	
LSU	17	845	52	43	
TEF	17	394	42	27	
ITS + LSU + TEF	17	1848	279	211	GTR + I + G

Abbreviations of the model: GTR + I + G (General Time Reversible model with invariant sites and gamma distribution).

## Data Availability

The original contributions presented in the study are included in the article/Supplementary Material, further inquiries can be directed to the corresponding authors.

## Supplementary Materials

The following supporting information can be downloaded at: https://www.mdpi.com/article/10.3390/jof11030180/s1, Figure S1: Maximum likelihood phylogeny of *Marquandomyces* inferred from the ITS dataset. Bootstrap values ≥ 50% are indicated at nodes. Asterisk denotes 100% bootstrap; Figure S2: Maximum likelihood phylogeny of *Marquandomyces* inferred from the LSU dataset. Bootstrap values ≥ 50% are indicated at nodes. Asterisk denotes 100% bootstrap; Figure S3: Maximum likelihood phylogeny of *Marquandomyces* inferred from the TEF dataset. Bootstrap values ≥ 50% are indicated at nodes. Asterisk denotes 100% bootstrap.
