# Supplementary material for: Five New Species of Marquandomyces (Clavicipitaceae, Ascomycota) from Asia
Source: J Fungi (Basel). 2025 Feb 25;11(3):180. doi: 10.3390/jof11030180 (PMC11943364; doi:10.3390/jof11030180)
Supplement: Supplementary file 1 [file jof-11-00180-s001.zip › Legends.pdf]

Figure S1. Maximum likelihood phylogeny of *Marquandomyces* inferred from the ITS dataset. Bootstrap values ≥ 50% are indicated at nodes. Asterisk denotes 100% bootstrap.

Figure S2. Maximum likelihood phylogeny of *Marquandomyces* inferred from the LSU dataset. Bootstrap values ≥ 50% are indicated at nodes. Asterisk denotes 100% bootstrap.

Figure S3. Maximum likelihood phylogeny of *Marquandomyces* inferred from the TEF dataset. Bootstrap values ≥ 50% are indicated at nodes. Asterisk denotes 100% bootstrap.
