# Supplementary figures and images for: Five New Species of Marquandomyces (Clavicipitaceae, Ascomycota) from Asia
Source: J Fungi (Basel). 2025 Feb 25;11(3):180. doi: 10.3390/jof11030180 (PMC11943364; doi:10.3390/jof11030180)

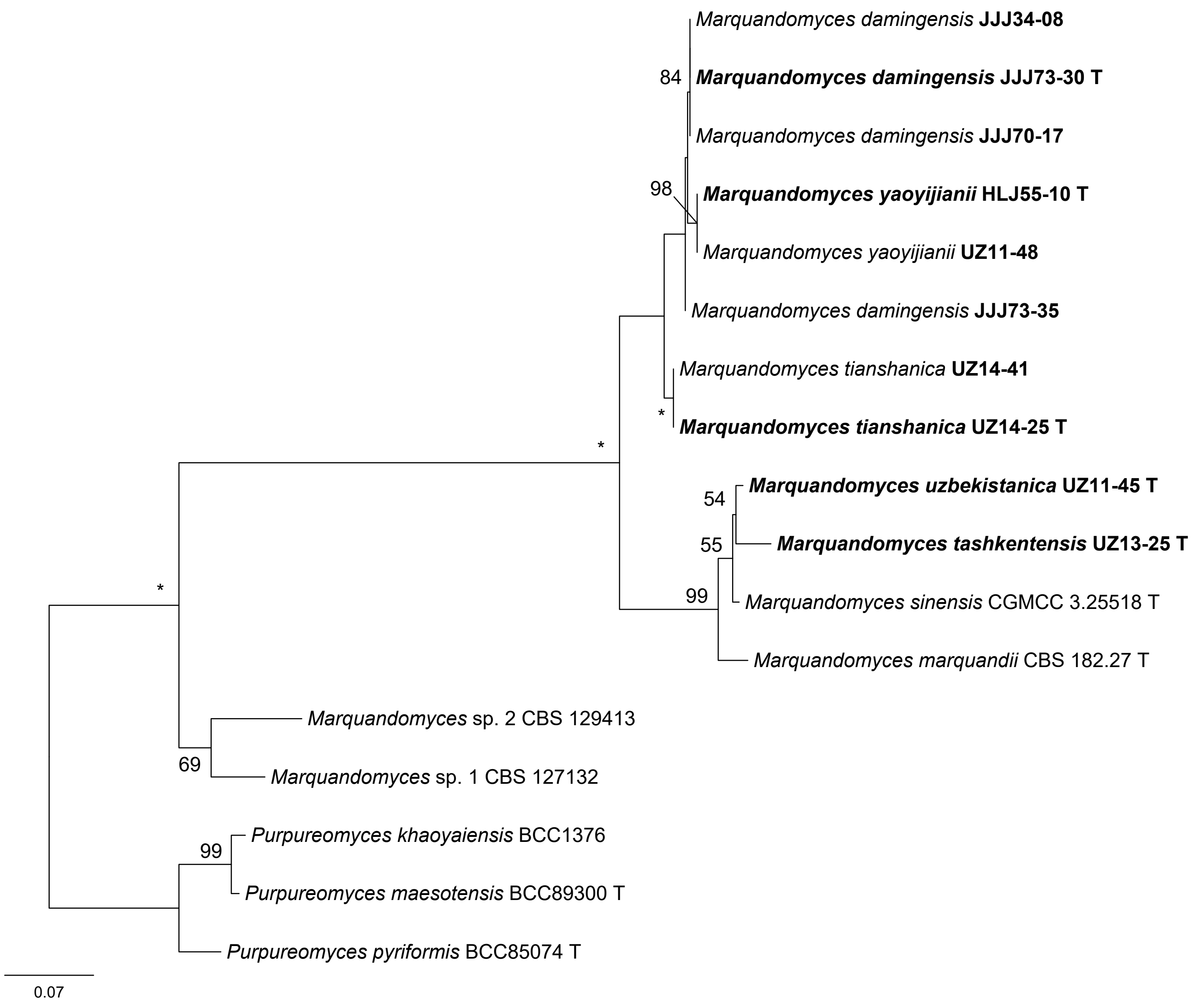

Supplement: Supplementary file 1 [file jof-11-00180-s001.zip › Figure S1 ITS.pdf]

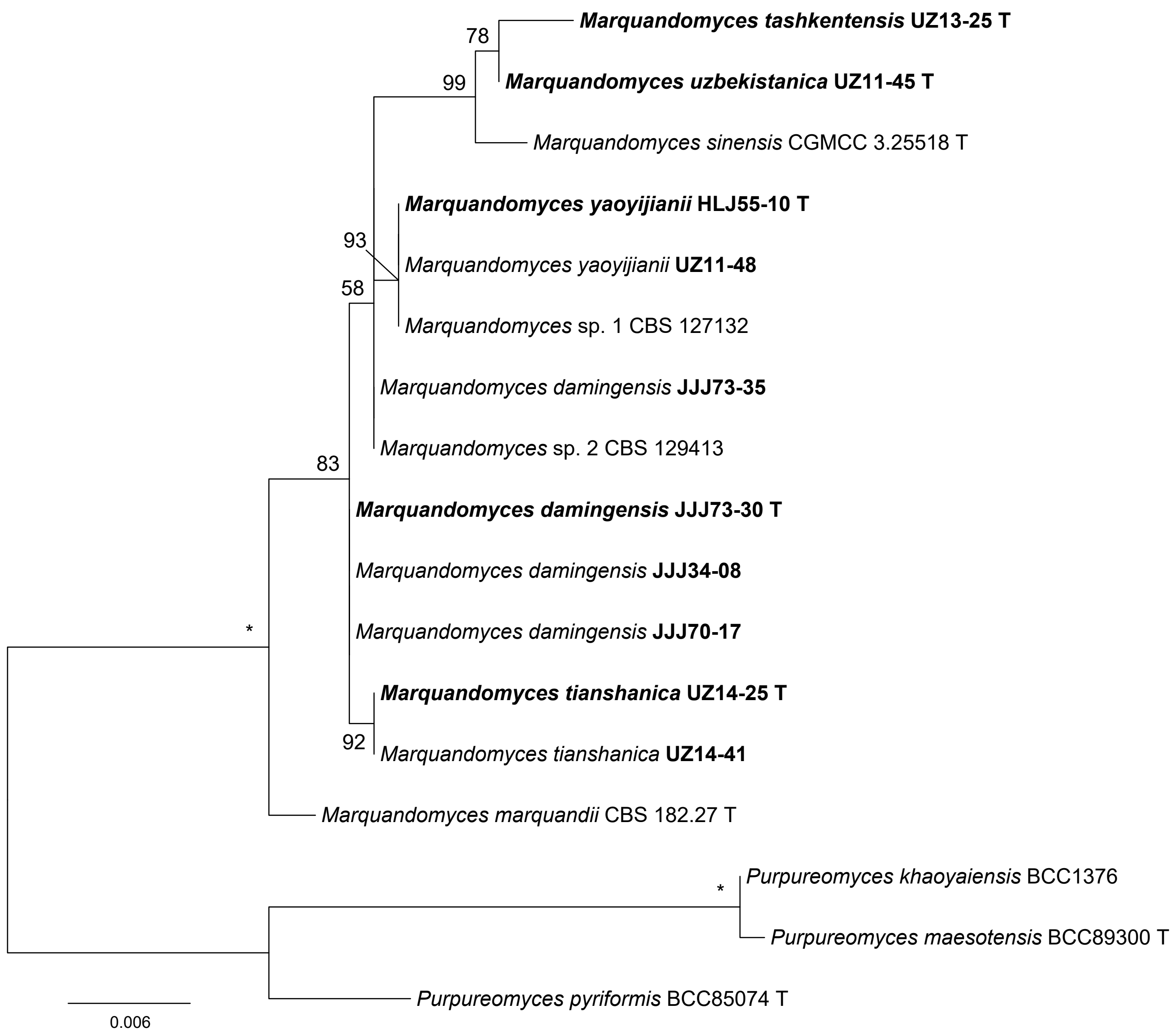

Supplement: Supplementary file 1 [file jof-11-00180-s001.zip › Figure S2 LSU.pdf]

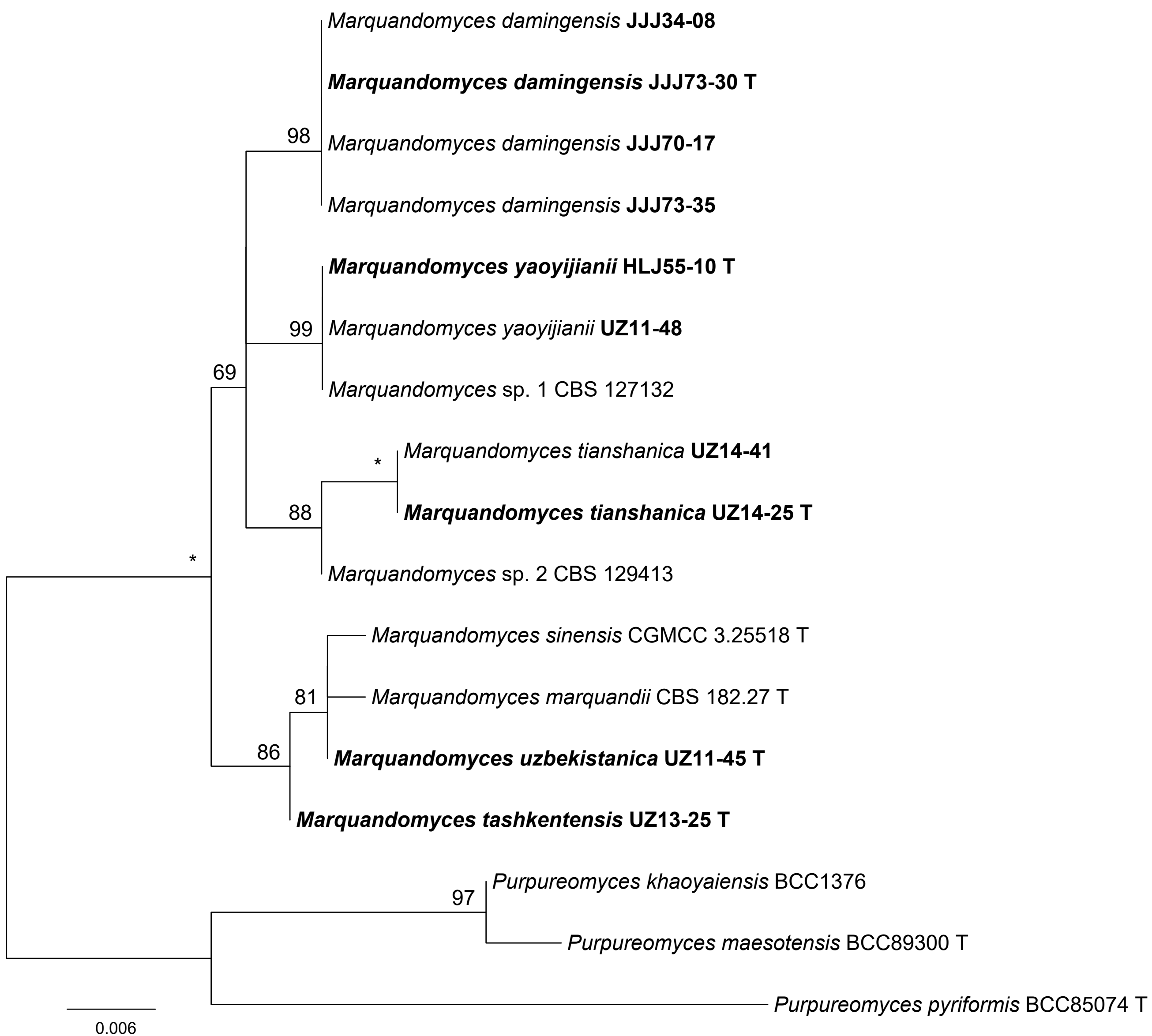

Supplement: Supplementary file 1 [file jof-11-00180-s001.zip › Figure S3 TEF.pdf]
